# Using simulation to introduce students and healthcare professionals to losses experienced by older adults: a pre-post analysis

**DOI:** 10.1186/s12909-024-05090-1

**Published:** 2024-01-29

**Authors:** Donna Prete, Linda Tamburri, Nicole Rolston, Marc Sturgill, Mary Bridgeman

**Affiliations:** 1 Robert Wood Johnson University Hospital-New Brunswick, 1 Robert Wood Johnson Place, New Brunswick, NJ 08901 USA; 2 Ernest Mario School of Pharmacy, Rutgers, The State University of New Jersey, 160 Frelinghuysen Road, Piscataway, NJ 08854 USA

**Keywords:** Older adult, Empathy, Simulation, Pharmacy, Nursing, Students

## Abstract

**Background:**

To introduce students and healthcare professionals to losses experienced by older adults and instill compassion among interprofessional learners, an interactive narrative simulation activity was developed and incorporated in clinical staff orientation and student professional course work. Narrative simulation allows learners to incorporate skills of examination, exploration, sharing, and reflection applied to simulated losses and lived experience of the older adult to promote empathy and understanding.

**Methods:**

A pre-post analysis was conducted to evaluate changes in self-reported empathy scores among nurses, pharmacists, student nurses and student pharmacists using the 20-item Jefferson Scale of Empathy©, Health Professional and Health Professional Student versions. The instrument was administered prior to and after narrative simulation participation.

**Results:**

A total of 152 students and 107 health care professionals completed both assessments. Median (interquartile range, IQR) post-simulation scores were significantly higher among nursing professionals [118.5 (112.25, 126.75) versus 126 (117, 132); *P* < 0.001; effect size 0.81] and nursing students [116 (107, 121) versus 119 (109, 126); *P* < 0.001; effect size 0.28], as well as pharmacy students [111 (101, 117) versus 116 (107.5, 125); *P* < 0.001; effect size 0.47]. Although a moderate effect size of 0.7 was observed for pharmacy professionals, there was no difference between pre- and post-activity empathy scores [117 (98, 137) versus 116 (101, 137); *P* = 0.16] for pharmacists participating in the narrative simulation exercise.

**Conclusions:**

A statistically significant change in self-reported levels of empathy, particularly for nurses, nursing students, and pharmacy students, was observed; results of this activity did not suggest a change in pharmacist self-reported empathy levels. This activity could be implemented by educators seeking to increase awareness of losses experienced by the older adult.

## Background

The projected growth of the older American population is unprecedented. According to U.S. Department of Health and Human Services, there were more than 55.7 million Americans aged 65 or older, representing 17% of the population, in the year 2020; more than one in every six Americans fall within this demographic group [1]. Population demographic growth has been increasing for more than a decade, with the population aged 65 or older increasing by 15.2 million since the year 2010. By 2040, it is estimated that there will be more than 80.8 million older adults living in the U.S., representing nearly 22% of the population [1]. Hence, as the older adult population continues to grow, healthcare providers must be prepared to provide person-centered care for the older adult that is respectful, responsive, empathetic and focused on the unique, heterogeneous, and often-complex, healthcare needs of this population.

While various definitions of empathy exist, empathy is the ability to understand, be aware of and sensitive to, and share the feelings and thoughts of another, encompassing recognizing and validating a person’s fears, anxiety, pain, and circumstances. (2–3) Empathy builds trust and represents an essential clinical competency for workers in the healthcare field [4]. As noted by Fjortoft and colleagues, empathy accounts for a significant aspect of the healthcare provider-patient relationship [5]. Empathy fosters a therapeutic partnership between the healthcare provider and the patient, contributes to the quality of care of the older adult patient and may facilitate improved patient outcomes and provide a positive patient experience [6]. There are different opinions as to the notion of whether empathy can be taught. According to Hojat, empathy is a cognitive attribute and not a personality trait; therefore, it can be taught [6]. Others believe that empathy is an unteachable phenomenon that can be facilitated through self-awareness, listening and experience [7]. Regardless of teaching or facilitating empathy, cultivating empathy is a life-long learning process necessary for healthcare providers.

Simulation, as a teaching strategy, is designed to provide experiential learning that facilitates the care providers’ development of clinical reasoning, psychomotor skills and reflection [8]. Moreover, the narrative pedagogy, an approach to teaching complex clinical care topics using storytelling and lived experience as part of simulation, incorporates examination, exploration, sharing, and reflection to promote empathy and understanding [9]. Therefore, when activities are developed for teaching, sensitization, and training specific to enhance the therapeutic relationship, it is thought that empathy generally increases. Current literature focuses on teaching empathy and caring behaviors in healthcare professional schools, and there is interest in evaluating levels of empathy among nurses, pharmacists and students in these healthcare professions; notably, specific educational strategies, interventions and best practices for increasing empathy in those providing care for the older adult are limited. The literature is rife with examples of workshops and simulation exercises designed to emulate the impacts of aging across the healthcare professions [10–12]. As examples of some of these strategies for developing empathy for older adults among students, Van Winkle described an interactive intervention with medical and pharmacy students focused on awareness of the challenges of aging and the need for empathy for this growing population [13]. The workshop noted short-term effects of the intervention; however, long-term effects were not sustained [13]. Research conducted by Sedaghati Kesbakhi and colleagues documented that oncology nurses studied in Iran showed positive attitudes towards empathy with oncology patients [14]. 

### Narrative simulation development

In 2015, the Narrative Geriatric Loss Simulation Exercise was incorporated as an orientation education tool for newly hired nurses and clinical care technicians (CCTs) at a large academic medical center in New Jersey. This 15-minute narrative, interactive, fictitious case simulation session, inspired by the game “*Into Aging”*, was designed to provide leaners with a personalized experience of age-related losses, including loss of physical function, independence, loved ones, and financial resources (Table 1) [15]. The simulation exercise was intended to simulate age-related physical changes, life events, and resulting emotions that occur with age, and the debriefing session was intended to guide learners in transforming the experience into empathetic caring practices. As this simulation garnered positive feedback from nurses and CCTs, it was decided to expand simulation participation to patient monitor orientation. Additionally, the exercise was further expanded to include student pharmacists as part of a palliative and end-of-life professional elective course with an emphasis on geriatrics.

After receiving positive, anecdotal, feedback from both nursing and pharmacist participants of the impact of simulation participation, investigators decided to measure the impact of the simulation on participant empathy prior to and after simulation participation.

**Table 1:** Narrative Geriatric Loss Simulation Exercise

On a piece of paper, write the following: • Your name • Your type of residence (apartment, condominium, house) • Your occupation (current or “dream job”) • Three people who are important in your life (family, friends) • Three treasured possessions (pet, photos, boat, jewelry, family memento, car) • One favorite hobby or pastime
Instructions to facilitators: After asking participants to record the information above, and distributing paper “money” to simulate personal finances, read the narrative below.
Imagine you are 60 years old. You have a full-time job that you enjoy and you have an active lifestyle. Your monthly income is sufficient to meet your needs with some additional cash for retirement savings. You like the neighborhood in which you live, and most of your family and friends live within a 30-minute drive. Your health is reasonably good– you have high blood pressure and mild diabetes, but they are both controlled with medication. You wear bifocal glasses for help with distance vision and reading. You are getting a little hard of hearing, but you can determine what people are saying if they face you when they talk. Because of dental problems, you recently had dentures (partial plate) made. Today you heard that someone very close to you is moving to another state 1,200 miles away. Due to the distance and the cost of air travel, you don’t expect to be able to visit them. **Cross one person off your list**.
You are now 64 years old and you notice you have been increasingly short of breath and very fatigued for the past several months. You visit your doctor and are told you have had a heart attack. After some time, you decide to cut down from full to part-time employment. You find yourself enjoying the additional time off. You have more free days to enjoy your hobbies. You had hoped to travel, but with the reduced income, you can no longer afford big trips. **Tear up one-third of your income**. You are content with trips to the beach in the summer and the Poconos in the fall.
At 66 you decide to retire– **cross off your occupation**. You begin receiving social security checks and draw money from your pension, but the income is still less than when you were working. **Tear up half of your income**.
Last week you turned 70 and your family had a surprise birthday party for you. You had a wonderful time at the party, but it made you think about many things. It is hard to believe you are 70; in your head you feel like you are still 21, but your body won’t do what you think you can do. Your heart disease makes your activity limited because of chest pain and shortness of breath. You have been told that your family is concerned about you. Since you no longer work, you find it hard to tell one day of the week from another, so sometimes they think you are confused. Your hearing loss is worsening, but this is embarrassing for you and rather than tell someone about the problem, you pretend you understand what people are saying when they speak to you. Your responses do not always make sense to others, and this further confirms your family’s belief that you are confused. One night, on the way to the kitchen, you slip on a small rug in the dark and you fall, breaking your arm. Your family decides it is no longer safe for you to live alone so they insist you live with one of them– **cross off your residence**. Instead of having an entire home to yourself, you will only have one bedroom and not much room to store your things– **cross off two possessions**.
Time goes by and you are adjusting to your new living arrangements. Others in the home seem so busy, but there is not much for you to do. You are 73 years old and no longer very active. No one asks for your help or your advice. You often think back to your youth. You did some exciting things when you were younger and had great success in your work, but your family and friends have already heard these stories several times and they don’t seem interested in them now. Since there is not much excitement in your life these days, you have little news to share with others and not many people call you anymore. You are no longer able to drive so you don’t get out unless someone takes you where you need to go, which most often is to the doctor. Yesterday, someone close to you passed away– **cross one person off your list**.
You are now 75 years old and have been hospitalized with a stroke. You have severe right-side paralysis; you cannot walk and cannot use your right arm (you are right-handed). It is hard to remember the names of all the staff. They all look the same, wear the same clothes, and there are several different people taking care of you every day. They wear name badges, but you cannot see them because your glasses are in the bedside table on the other side of the room. To get the staff’s attention when you need help, you wave at them as they walk by your room. Some ignore you as they rush off to complete their tasks. Others smile, wave back and keep on walking. Many staff members do not call you by your name, instead you are called: (Provide names to the participants such as Pops, Grandma, Dear, Honey, Sweetie, Sugar, Cookie, Sweetie Pie, Room 12 bed 1; Room 5 bed 2, the CVA, or no name at all). **Cross off your name and replace it with the nickname given to you.**
Your meal tray has arrived. The stroke has made it difficult for you to swallow and you are given pureed food. You try to feed yourself with your left hand and usually make a mess of the tray and your gown. You don’t get much food to your mouth, but it doesn’t matter; you’re not very hungry most of the time. Your health care team decides you need rehab and long-term care. Arrangements are made for you to go to a nursing home. **Cross off one possession and one hobby. Hand over your remaining income.**
Instructions to facilitators: Direct the discussion to include addressing issues including independence; dignity; making a contribution/feeling valued by society and family; identity. Review the debriefing questions (below) with participants upon activity conclusion. Debriefing Questions: 1. How did this feel? 2. Did you feel like you were being treated unfairly because of your age? When? 3. Which was the hardest loss to endure? 4. At what point did life seem to be very difficult? Why? 5. Is there anything that would have been a help for you? 6. What are the most important take-away messages to apply to your work as a CCT/nurse/pharmacist/student professional?

## Methods

The objective of this study was to evaluate the impact of participation in a narrative simulation designed to convey the losses experienced by older adults. Participants in the simulation exercise were invited to partake in a pre- and post-simulation empathy assessment. The paper-based survey was distributed to various groups of learners, including nurses, student nurses, pharmacists, and student pharmacists, and was completed immediately prior to and immediately after simulation participation. A validated instrument developed to measure empathy in the context of patient care among healthcare providers and students, the Jefferson Scale of Empathy© (Health Professional (HP) and Health Professional Student (HPS) Versions) was used to measure self-reported aggregate empathy scores amongst the various participants invited to partake in this exercise and study [16, 17]. Survey participants were asked to provide demographic information and to self-evaluate their level of empathy using a Likert scale (1 = strongly disagree, 7 = strongly agree) on this 20-item survey instrument, where half of the survey items are positively worded and directly scored on Likert weights and the other half of the items are negatively worded and reverse scored and higher scores indicate more empathic behavioral orientation. Permission to use the questionnaire, as well as institutional review board approval, were obtained prior to initiation of data collection; all participants consented to study participation. Five additional questions regarding the quality and impact of the activity were included on the post-simulation assessment to evaluate the activity’s ability to improve the professional or student’s ability to recognize losses of the older adult and identify the various needs of the older adult; to identify the value of the activity in professional development; to assess the activity’s length to complete; and whether the feelings from this simulation might be incorporated into clinical care for the geriatric population. No formal statistical analysis was completed on these items; descriptive statistics were reported. This study was granted exempt status by the University’s Institutional Review Board; participants provided informed consent prior to study participation.

### Statistical methods

All Likert scale responses were treated as ordinal data. The primary study outcome (pre- versus post-activity empathy scores in both students and healthcare professionals) was analyzed using a Wilcoxon signed rank test. Effect sizes were given as the probability of superiority (${\widehat{P}}_{a>b}$) using the dominance statistic, or the number of times the post-activity score was greater than the pre-activity score, divided by the total number of matched scores minus any ties (${\widehat{P}}_{a>b}=\frac{{n}_{+}}{N}$) [18]. Cohen et al. defines a small effect as 0.2, a moderate effect as 0.5, and a large effect as 0.8 [19]. Secondary outcomes (a comparison between student and healthcare professional empathy scores both pre- and post-activity, sex differences in empathy scores both pre- and post-activity, and differences in empathy scores between practicing nurses and pharmacists both pre- and post-activity) were analyzed using a Mann-Whitney Rank Sum test. Effect sizes were calculated as the probability of superiority (${\widehat{P}}_{a>b}$), or the Mann-Whitney U statistic divided by the product of the two sample sizes (${\widehat{P}}_{a>b}=\frac{U}{{n}_{a}{n}_{b}}$) [20]. All analyses were carried out using SigmaPlot 12.0 (Systat Software, San Jose, CA). A *P* value < 0.05 was considered statistically significant for all analyses.

## Results

A total of 152 health professional students and 107 health care professionals participated in the narrative simulation exercise and completed both pre- and post-simulation assessments. Table 2 includes a summary of the demographics of the study population. The results of a comparison of pre- versus post-activity empathy scores in students and healthcare professionals is given in Table 3. Median (IQR) post-simulation scores were significantly higher in both nursing professionals and nursing students as well as pharmacy students. Although a moderate effect size of 0.7 was observed for pharmacy professionals, there was no difference between pre- and post-activity empathy scores.

**Table 2:** Demographic characteristics of the study participants

	Age (years) No. (%)	Sex No. (%)
**Pharmacists**	21–30, 3 (27.3) 31–40, 3 (27.3) 41–50, 3 (27.3) 51–60, 2 (18.2)	Male, 2 (18.2) Female, 7 (63.6) Unknown, 2 (18.2)
**Pharmacy students**	19–21, 2 (3.08) 22–24, 56 (86.1) 25–27, 5 (7.69) 28–30, 1 (1.54) 34–36, 1 (1.54)	Male, 20 (30.8) Female, 40 (61.5) Unknown, 5 (7.69)
**Nurses**	21–30, 48 (50.0) 31–40, 29 (30.2) 41–50, 15 (15.6) 51–60, 4 (4.17)	Male, 16 (16.7) Female, 79 (82.3) Unknown, 1 (1.04)
**Nursing students**	19–21, 2 (2.33) 22–24, 40 (46.5) 25–27, 20 (23.3) 28–30, 11 (12.8) 31–33, 3 (3.49) 34–36, 3 (3.49) 37–39, 2 (2.33) 40–42, 3 (3.49) 43–45, 1 (1.16) 46–48, 1 (1.16)	Male, 15 (17.2) Female, 61 (70.1) Unknown, 11 (12.6)
**Other**	21–30, 1 (100)	Female, 1 (100)
**Other students**	25–27, 1 (100)	Female, 1 (100)

**Table 3:** A comparison of pre- and post-simulation empathy scores in both students and healthcare professionals

Profession	Pre-simulation [Median (IQR)]	Post-simulation [Median (IQR)]	Effect size	*P* value
Nurse (*n* = 96)	118.5 (112.25, 126.75)	126 (117, 132)	0.81	< 0.001
Pharmacist (*n* = 11)	117 (98, 137)	116 (101, 137)	0.70	0.16
**Total (Nurse and Pharmacist)** (*n* = 107)	118 (111, 127)	126 (116, 132)	0.80	< 0.001
Nursing student (*n* = 87)	116 (107, 121)	119 (109, 126)	0.75	< 0.001
Pharmacy student (*n* = 65)	111 (101, 117)	116 (107.5, 125)	0.84	< 0.001
**Total (Nursing and Pharmacy Student)** (*n* = 152)	112 (106, 119)	117.5 (109, 125.75)	0.79	< 0.001

A comparison between student and healthcare professional empathy scores both pre- and post-activity is given in Table 4. Median (IQR) empathy scores were significantly higher in nursing professionals than in nursing students, both pre-activity and post-activity. There were no differences between pharmacy professional and pharmacy student empathy scores either pre-activity or post-activity.

**Table 4:** A comparison between student and healthcare professional empathy scores pre- and post-simulation

Study period	Profession	Student [Median (IQR)]	Professional [Median (IQR)]	Effect size	*P* value
Pre-activity	Nursing	116 (107, 121); *n* = 87	118.5 (112.25, 126.75); *n* = 96	0.37	0.00244
	Pharmacy	111 (101, 117); *n* = 65	117 (98, 137); *n* = 11	0.36	0.1556
Post-activity	Nursing	119 (109, 126); *n* = 87	126 (117, 132); *n* = 96	0.34	0.0002
	Pharmacy	116 (107.5, 125); 65	116 (101, 137); *n* = 11	0.40	0.27527

A pre- and post-activity comparison of empathy between nursing and pharmacy professionals, as well as nursing and pharmacy students, is given in Table 5. The only observed difference occurred in pre-activity scores between nursing and pharmacy students.

**Table 5:** A comparison of nursing professional and nursing student versus pharmacy professional and pharmacy student empathy scores pre- and post-simulation

Study period	Profession	Nursing [Median (IQR)]	Pharmacy [Median (IQR)]	Effect size	*P* value
Pre-activity	Professionals	118.5 (112.25, 126.75); *n* = 96	117 (98, 137); *n* = 11	0.47	0.723
	Students	116 (107, 121); *n* = 87	111 (101, 117); *n* = 65	0.40	0.031
Post-activity	Professionals	126 (117, 132); *n* = 96	116 (101, 137); *n* = 11	0.46	0.644
	Students	119 (109, 126); *n* = 87	116 (107.5, 125); *n* = 65	0.45	0.309

A comparison of empathy scores by sex, in both healthcare professionals and students both pre- and post-activity is given in Table 6. Pre-activity median (IQR) empathy scores were significantly higher in females than in males, both among healthcare professionals and healthcare students. Post-activity scores remained significantly higher in female students as compared to male students, but there was no difference between male and female healthcare professionals.

**Table 6:** Student and healthcare professional empathy scores by sex pre- and post-simulation

Study period	Profession	Male [Median (IQR)]	Female [Median (IQR)]	Effect size	*P* value
Pre-activity	Professionals	110.5 (103, 118.25); *n* = 18	122 (113, 127); *n* = 87	0.29	0.006
	Students	108 (96, 120); *n* = 35	115 (108, 121); *n* = 102	0.36	0.013
Post-activity	Professionals	122.5 (105, 131); *n* = 18	126 (117, 133); *n* = 87	0.38	0.121
	Students	114 (95, 122); 35	120 (113, 127); *n* = 102	0.35	0.007

A post-activity program assessment was additionally performed. Regardless of profession or student or professional participant, all individuals surveyed reported agreement or strong agreement that the simulation improved their ability to recognize life losses experienced by older adults; improved their ability to identify needs of the older adult; was valuable and of an appropriate length. Notably, there was agreement and strong agreement among participants that the feelings evoked by this simulation would likely be incorporated into their management of older adult patients.

## Discussion

Improving care for older adults and preparing a workforce to meet the clinical care needs of the older adult population remain global health imperatives. Numerous educational approaches to simulate the experience of aging have been described previously; however, the authors of this manuscript believe this is the first description of a narrative simulation exercise, based on a fictitious patient lived experience, to enhance empathy toward older adults across nursing and pharmacy professionals and students. Although aging sensitization games, exercises, and simulation-based activities are not uncommon in health professional training, including for student nurses and pharmacists, this activity is unique in its narrative simulation format and delivery, which results in minimal equipment cost, set up time and space requirements for delivery [10, 12–13, 21−24].

In evaluating the aggregate pre- and post-simulation median scores, a statistically significant and likely clinically meaningful difference in nurse, total healthcare professional and pharmacy student participant empathy scores were observed; moderate effects on pharmacist, nursing student and total healthcare student empathy scores were observed which may not be clinically meaningful. While examining professional designation and whether student or professional participant, nurses and nursing students generally reported higher pre-activity median empathy scores and significantly higher post-activity empathy scores. Additionally, pharmacy students were observed to experience significant increases in self-reported empathy post-simulation participation. While pharmacists did not experience a statistically significant change in post-workshop empathy scores, it should be noted that the small number (*n* = 11) of participants in this group may reflect a lack of power to detect change.

In evaluating participant subgroups, statistically significant differences in median empathy scores between students and healthcare professionals were observed, with nurses having higher self-reported empathy scores both prior to and after simulation participation. While higher self-reported empathy scores at baseline among nurse participants may be reflective of the effect of career experience on one’s empathy, it is interesting to note that nurses, who have greater empathy education and more extended daily bedside patient interactions, were additionally observed to have a greater change in empathy scores in the context of this exercise, Notably, both nurses and nursing students reported significant increases in self-reported empathy post-simulation participation, although statistically significant differences between pharmacist and student pharmacist self-reported empathy were not observed. The lack of observed difference, particularly among pharmacists, may be reflective of limited power and small numbers of participants to detect such a change.

The influence of biological sex on one’s empathetic ability remains an elusive point of ongoing scientific debate [25]. In our study, significant differences based on sex were observed, with female students and professionals generally reporting greater empathy at baseline. Differences in empathy according to sex disappeared after simulation participation for the health professionals but persisted for student participants.

The limitations of this study must be considered. The subjective nature of empathy results in difficulty in quantitatively measuring changes in scores, or the likelihood that a single exercise would influence a professional’s empathy over time. Empathy is very individualized based on one’s own life experiences and perceptions, and factors such as age, years in practice, and sex may influence self-reported empathy. Furthermore, although investigators demonstrated a significant impact on participant self-reported empathy scores in the short-term, researchers are unable to identify from this methodology whether the difference in pre- and post-simulation self-reported empathy scores translates into improved care or compassion in interacting with older adult patients and if effects are sustained. A longitudinal study design would provide a better assessment of the sustainability of the impact of the simulation or if the behavior becomes habitual. It must also be noted that researchers must be well versed in the scoring of the Jefferson Scale of Empathy© tool. Half of the items are positively worded and directly scored, while the other half of the items are negatively worded and reverse scored. Of note, both the practicing health care professional version and the health professions student version of the instrument were utilized for this study. Investigators should be aware that there is also a licensing fee to utilize the instrument.

It should be noted that, regarding activity quality and impact, all individuals surveyed, regardless of professional experience or role, reported agreement or strong agreement that the simulation improved their ability to recognize life losses experienced by older adults; improved their ability to identify needs of the older adult; was valuable and of an appropriate length. Learners believed this impact would be likely to translate into their ability to provide care for the older adult population.

## Conclusions

A significant change in self-reported levels of empathy was observed in most simulation participants. Educators and clinician alike seeking to increase awareness of losses facing the older adult, or interested in incorporating simulation as part of a geriatrics-based training program, could easily replicate this activity. This activity has gained traction throughout our organization, including being shared with a system-wide Geriatrics Collaborative and incorporated in orientation onboarding seminars for new hires in clinical practice throughout the system. This is an important exercise that could be incorporated into professional training curricula as well to support patient-centered care delivery for the older adult.

## Data Availability

The datasets used and analysed during the current study are available from the corresponding author on reasonable request.

## Abbreviations

CCT: Clinical Care Technician
RN: Registered Nurse
HPS: Health Professional Student
HP: Health Professional
SPSS: Statistical Package for the Social Sciences
